# Tensile Properties of Cryorolled Cu/Al Clad Sheet with an SUS304 Interlayer after Annealing at Various Temperatures

**DOI:** 10.3390/ma17164065

**Published:** 2024-08-15

**Authors:** Yanni Xuan, Jing Li, Haitao Gao, Hailiang Yu

**Affiliations:** 1 School of Energy and Power Engineering, Changsha University of Science and Technology, Changsha 410114, China; xuanyanni@csut.edu.cn; 2 State Key Laboratory of Precision Manufacturing for Extreme Service Performance, Light Alloys Research Institute, Central South University, Changsha 410083, China; 183701039@csu.edu.cn (J.L.); yuhailiang@csu.edu.cn (H.Y.)

**Keywords:** Cu/Al clad sheet, SUS304 interlayer, annealing treatment, cryorolling, tensile properties

## Abstract

This paper investigates the tensile properties and microstructures of Cu/Al clad sheets with an SUS304 interlayer after cryorolling and subsequent annealing and compares them with hot-rolled samples. The experimental results show that the inhibition of dynamic recovery by cryorolling enables the Cu/Al clad sheets to achieve a tensile strength of 302 MPa. After annealing, the tensile strength sharply drops to 159 MPa, while the elongation recovers to 29.0%. Compared with hot-rolled samples, the tensile strength of cryorolled samples is increased by 13.1% due to the effect of fine-grain strengthening. During the annealing process, the cryorolled samples exhibit improved elongation under a comparable strength with the hot-rolled samples, profiting from the higher degree of recrystallization and a higher proportion of annealing twins. The tensile properties of Cu/Al clad sheet with an SUS304 interlayer are strengthened by cryorolling and subsequent annealing, providing a new method for the fabrication of high-performance Cu/Al clad sheets.

## 1. Introduction

Cu/Al clad sheets are extensively used in many important fields, such as aerospace and electronic communication. [1,2,3]. Traditional methods for producing Cu/Al clad sheets include roll bonding [4], cast-rolling bonding [5], and explosive bonding [6]. Cryogenic process technology is an emerging technique utilized in the fabrication of high-performance metallic materials [7]. Wang et al. [8] investigated the microstructure, texture, and mechanical properties of AA1060 sheets processed by accumulative roll bonding (ARB) at cryogenic temperatures. They found that initial cube texture was transformed into r-cube texture with increasing ARB cycles. Takagawa et al. [9] successfully enhanced the mechanical properties of the Cu-Ni-Si-Zr alloy without compromising its electrical conductivity through integrating ARB, cryorolling, and aging treatment. The tensile strength of the cryorolled clad sheets was 36.7% higher than those produced by cold-roll bonding. Furthermore, Liu et al. [10] adopted cryorolling to improve the interfacial bonding strength of Al/Ti/Al clad sheets. They found that cryorolling could improve the interfacial mechanical locking strength. Therefore, studying the mechanical properties of metallic clad sheets during cryorolling is of great significance.

Annealing treatment significantly impacts the microstructure and comprehensive performance of metallic clad sheets [11,12]. As a result of the powerful chemical affinity between Cu and Al, intermetallic compounds (IMCs) may form at the bonding interface [13]. Consequently, extensive studies have been conducted to control the Cu-Al IMCs. During annealing, Tayyebi et al. [14] discovered that the formation sequence of the IMC layers was Al_2_Cu, Al_4_Cu_9_, and AlCu, and that the growth of IMCs was mainly controlled by diffusion. Pelzer et al. [15] established a mathematical relation between IMC thickness and annealing parameters. In a similar vein, Lee et al. [16] pointed out that Cu-Al IMC thickness can be regulated through diffusion mechanisms. Mao et al. [5] indicated that the Cu-Al IMC thickness of 250 °C-annealed clad sheets can be controlled at a nanometer level, with a peeling strength of 39 N/mm. Thus, regulating the microstructure of IMCs is crucial for achieving high-performance Cu/Al clad sheets. However, previous studies primarily focused on the interfacial bonding strength of metallic clad sheets, with limited research on their tensile properties, especially those clad sheets annealed after cryorolling.

Our previous research revealed that the bonding strength of Cu/Al clad sheets can be significantly improved by introducing a 304 stainless steel (SUS304) interlayer [17]. To explore the tensile properties of cryorolled clad sheets containing an interface interlayer after annealing at various temperatures, Cu/Al clad sheets with an SUS304 interlayer are fabricated using a combination of hot rolling and cryorolling, and the influence of annealing temperature on the tensile properties of Cu/Al clad sheet was investigated. Additionally, the differences in tensile properties of hot-rolled and cryorolled samples are systematically compared.

## 2. Materials and Methods

**Figure 1:**
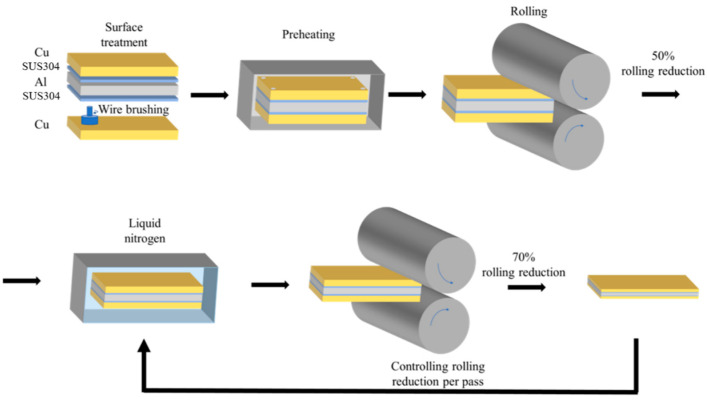
Schematic illustration of cryorolled Cu/Al clad sheets with an SUS304 interlayer.

The initial matrix materials are fully annealed T2 Cu (99.9%) sheet and 1060 Al (99.6%) sheets, both with a thickness of 1 mm. Cold-rolled SUS304 foil (30 µm in thickness) is selected as the interlayer material [17]. The mechanical properties of Cu, Al, and SUS304 foil are provided in Table 1. Prior to roll bonding, steel wire brushes were used to remove the oxide layers from the surfaces of Cu and Al sheets. Figure 1 illustrates the detailed preparation process of the cryorolled Cu/Al clad plate with an SUS304 interlayer. First, the Cu sheet, SUS304 foil, and Al sheet were assembled as a clad slab. The starting dimension of the assembled clad slab was 50 mm (length) × 40 mm (width). Next, the clad slab was hot-rolled (preheated at 450 °C for 1 min) to a 50% reduction, allowing for an initial bonding of the interfaces. Subsequently, cryorolling was performed with the following specific steps: To ensure heat conduction and minimize the impact of deformation heat during cryorolling, the single-pass reduction was set as 0.02 mm, and the rolling speed was adjusted to 5 m/min. After each pass, the sheet was re-immersed in liquid nitrogen for 5 min, until a total rolling reduction of 70% (final thickness of 0.6 mm) was achieved after 20 passes.

To investigate the influencing mechanisms of cryorolling on the tensile properties of clad sheets, a clad sheet with a 70% reduction achieved through single-pass hot rolling was specifically used as the control group. Subsequently, both the cryorolled samples and hot-rolled samples underwent annealing treatment at 200 °C, 300 °C, and 400 °C for 30 min. According to the standard of ISO 6892 [18], samples with a gauge size of 13 mm in length, 2.5 mm in width, and 0.4 mm in thickness were cut from the rolled Cu/Al clad sheets, ensuring that the direction of the parallel section of the tensile specimen is along the rolling direction. Room-temperature quasi-static uniaxial tensile tests (ISO 6892) were conducted using a Shimadzu AGS-X 10 kN tensile testing machine (Shimadzu, Kyoto, Japan) at a speed of 1 mm/min. Three specimens were tested for each condition to minimize the impact of errors. To study the microstructure of the Cu/Al clad sheets, Tescan MIRA3 LMU FE-SEM (Brno, Czech Republic) was adopted and electron backscatter diffraction (EBSD) experiments were performed using an Oxford Nordlys Max 3 system (Oxford Instruments, Abingdon, UK).

## 3. Results and Discussion

### 3.1. Microstructure Evolution of Cryorolled Samples during Annealing

**Figure 2:**
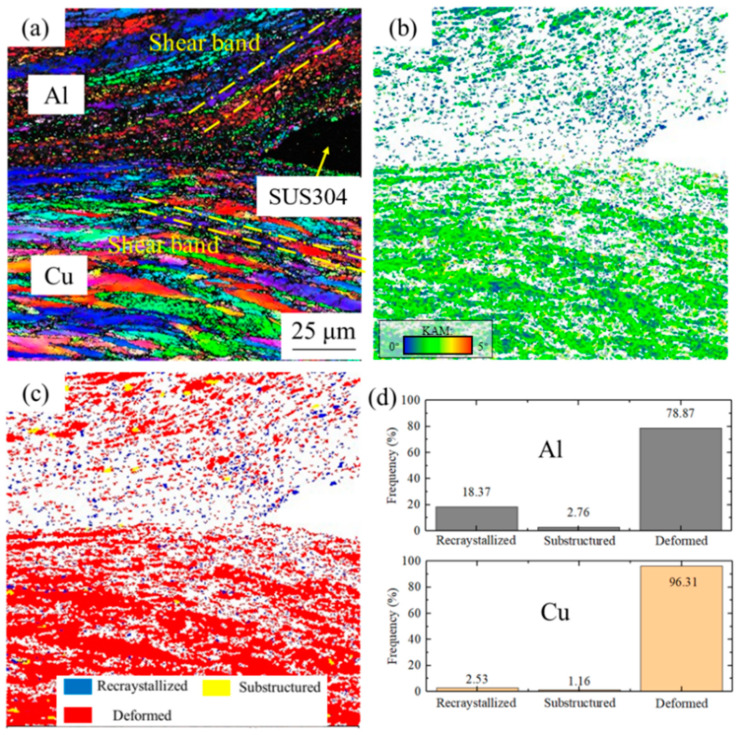
EBSD results of the cryorolled specimen: (**a**) IPF map. (**b**) KAM results: the green and blue colors represent the Kernel average misorientation. (**c**) Recrystallization degrees: blue stands for the recrystallized grains, yellow stands for the substructured grains, and red stands for the deformed grains. (**d**) Structural composition.

Figure 2 shows the microstructure of the cryorolled sample and the distribution results of its recrystallization degree. In Figure 2a, both the Cu and Al layers have undergone severe plastic deformation, and the grains exhibit a typical strip shape caused by rolling. Furthermore, additional plastic deformation is introduced by shear bands at the edges close to SUS304 fragments, resulting in more severe grain refinement [19]. The numerous regions difficult to analyze by EBSD in Figure 2a and the KAM results in Figure 2b both indicate a significant stress distribution within Cu/Al clad sheets after cryorolling.

In Figure 2c, the recrystallization distribution map of the cryorolled clad sheet reveals that deformed grains essentially fill the entire Cu and Al layers, with partial recrystallized grains and substructured grains only observed in the Al layer. These randomly distributed recrystallized grains and substructured grains in the Al layer are primarily due to the layer’s lower melting point and higher stacking-fault energy, which facilitate dynamic recrystallization and dynamic recovery during cryorolling. Figure 2d shows the analyses of the structural compositions in both Cu and Al layers. The proportion of deformed grains in the two layers is the largest, which corresponds to the description in Figure 2c. Specifically, in the Al layer, the fraction of recrystallized grains, substructures, and deformed grains are 18.37%, 2.76%, and 78.87%, respectively. In the Cu layer, the proportions are 2.53% for recrystallized grains, 1.16% for substructures, and 96.31% for deformed grains. Due to the relatively low recrystallization temperature, more recrystallized grains are formed during hot rolling. Therefore, there are much more deformed grains in the Cu layer than the Al layer for the cryorolled sample.

**Figure 3:**
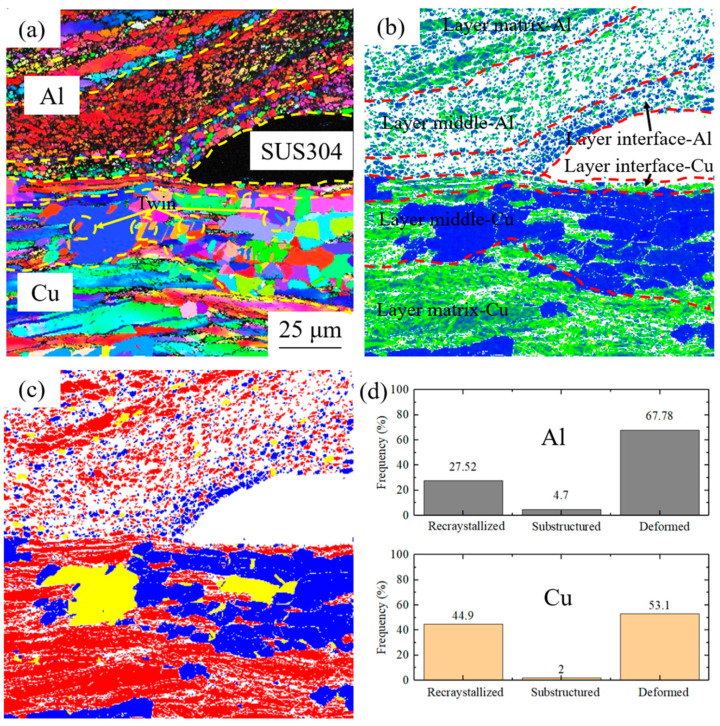
EBSD results of 200 °C-annealed cryorolled specimen: (**a**) IPF map, (**b**) KAM results, (**c**) recrystallization degree, and (**d**) structural composition.

Figure 3 presents the microstructure and recrystallization degree of the cryorolled sample after annealing at 200 °C. A notable difference in microstructure are observed between the Cu and Al layers (Figure 3a), both exhibiting a distinct three-layer distribution. On the Al side, the grains within the layer adjacent to the interface (Layer: interface-Al) show a slight degree of growth, with increased misorientation angles between adjacent grains, indicating a tendency towards random orientation. This primarily resulted from the additional shear deformation introduced by the addition of the SUS304 interlayer during cryorolling, which results in higher stored energy and weaker resistance to recrystallization in this region. The densely packed grain boundaries serve as favorable sites for recrystallization, leading to higher nucleation and growth rates. Consequently, recrystallization occurs readily in this region, producing finer recrystallized grains. The middle layer (Layer middle-Al) largely retains its cryorolled state, with grains exhibiting clear rolled lamellar structures and a high density of grain boundaries. In contrast, the layer adjacent to the matrix (Layer matrix-Al) contains some large-sized grains with relatively fewer internal grain boundaries. On the copper side, most grains in the layer adjacent to the interface (Layer interface-Cu) retain their rolled lamellar shape. Within the middle layer (Layer middle-Cu), grain sizes increase significantly, and the grain shapes approach equiaxed crystals, with numerous annealing twins present. In the layer adjacent to the matrix (Layer matrix-Cu), the overall grain sizes are larger, but they do not resemble the equiaxed grains found in the middle layer, instead retaining a shape closer to the rolled lamellar structure.

In the KAM results (Figure 3b), the layer on the Al side adjacent to the interface primarily comprises regions with relatively low strain. The middle layer is densely populated with high-strain regions and unresolved areas, both indicating significantly higher stress distributions in this region. In the layer adjacent to the matrix, the strain remains high but is accompanied by a small number of low-strain regions. On the copper side, the layer adjacent to the interface exhibits high strain, while the majority of the middle layer undergoes stress relief, dominated by low-strain regions. In contrast, the layer adjacent to the matrix still contains regions with high strain, interspersed with a few low-strain areas.

In the recrystallization distribution map, the Al side is predominantly composed of deformed grains. Within the thin layer adjacent to the interface, recrystallization of grains is evident, which explains the observed random orientation of equiaxed grains and low-strain distribution in this layer. In contrast, the middle and adjacent matrix layers exhibit majority of deformed grains interspersed with a minor fraction of substructured grains, likely attributed to incomplete recovery during low-temperature annealing. The layer on the Cu side adjacent to the interface comprises deformed grains, while recrystallized grains and substructured grains emerge in the middle layer. The adjacent matrix layer is dominated by deformed grains with a low presence of recrystallized grains. Statistical analysis of structural composition within the Cu and Al layers (Figure 3d) reveals that in the Al layer, recrystallized grains account for 27.52%, substructures for 4.7%, and deformed grains for 67.78%. In the Cu layer, the corresponding proportions are 44.9% for recrystallized grains, 2% for substructures, and 53.1% for deformed grains. For the cryorolled samples (Figure 2d), the more deformed grains in Cu layer suffer more accumulate defects, decreasing the energy barrier for recrystallization nucleation and promoting the formation of more recrystallized grains in Cu layer during low-temperature annealing.

**Figure 4:**
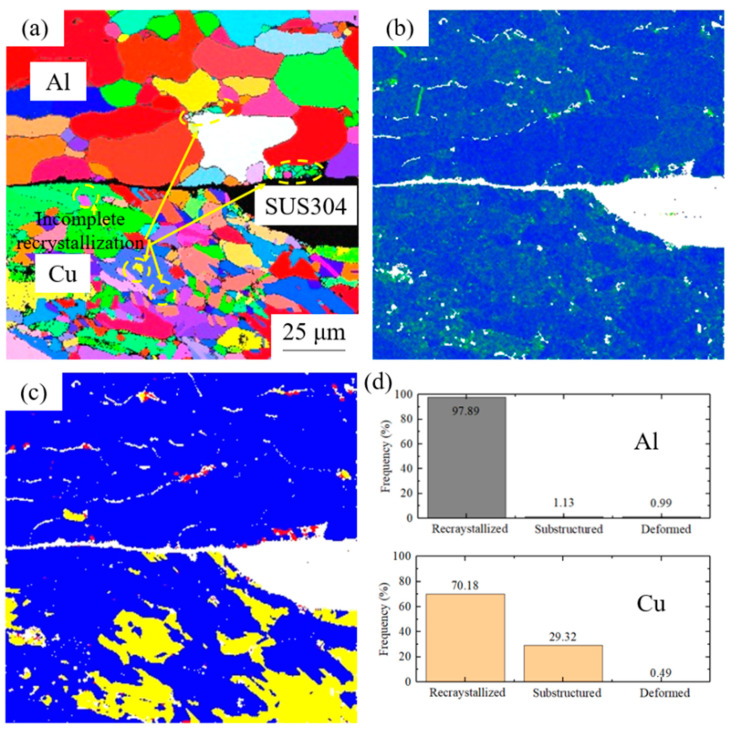
EBSD results of 300 °C-annealed cryorolled specimen: (**a**) IPF map, (**b**) KAM results, (**c**) recrystallization degree, and (**d**) structural composition.

Figure 4 presents the microstructural organization and recrystallization degree of the cryorolled specimen after annealing at 300 °C. As evident from the IPF map in Figure 4a, significant grain growth is seen in both the Cu and Al layers, with the rolling texture virtually eliminated. A random orientation characteristic of recrystallization occurs, and the layered structure in Figure 3 is absent after annealing at 300 °C. The recovery and softening of Cu and Al layers are the primary reasons for the rapid decline in the tensile strength of the clad sheet [20]. At the interface of the cryorolled clad sheet, the refined microstructure is largely absent, with only trace amounts of fine grains retained in the contact regions between the SUS304 interlayer and base metals. IMCs are present at the contact regions of Cu and Al layers.

The KAM results in Figure 4b indicate that stress in both Cu and Al layers is effectively relieved after annealing at 300 °C, with no significant stress concentration areas observed in either matrix. Figure 4c reveals that the deformed microstructure is largely absent, with the Cu and Al layers mainly composed of recrystallized grains and substructures. Only a small number of fine deformed grains are observed at the grain boundaries of recrystallized grains within the Al layer. Within the substructured grains of the Cu layer, grains of smaller sizes and varying orientations can be seen, which are attributed to incomplete recrystallization. The structural composition of the Cu and Al layers, presented in Figure 4d, shows that in the Al layer, recrystallized grains account for 97.89%, substructures for 1.13%, and deformed grains for 0.99%. In the Cu layer, the corresponding proportions are 70.18% for recrystallized grains, 29.32% for substructures, and 0.49% for deformed grains. Under 300 °C annealing, the recrystallization degree of the Al layer is much higher than that in the Cu layer due to the comparatively low recrystallization temperature, which is obviously different from the 200 °C-annealed samples (Figure 3d).

**Figure 5:**
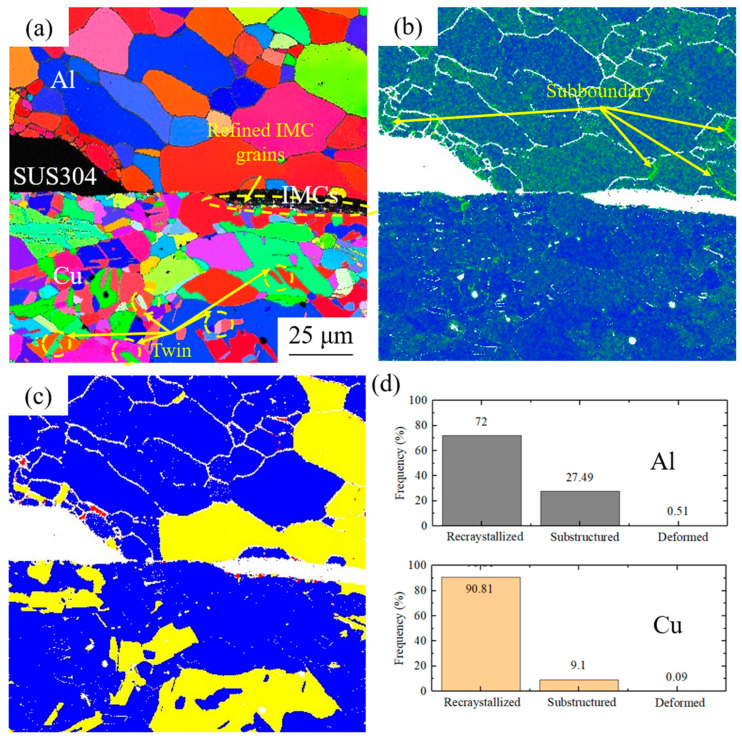
EBSD results of 400 °C-annealed cryorolled specimen: (**a**) IPF map, (**b**) KAM results, (**c**) recrystallization degree, and (**d**) structural composition.

Figure 5 illustrates the microstructural organization and recrystallization degree of the cryorolled specimen after annealing at 400 °C. The grains in both the Cu and Al layers undergo further growth, with a notable presence of annealing twins within the Cu layer and an obvious increase in IMC thickness. As depicted in the KAM results in Figure 5b, further stress release is observed, with a relatively dense stress distribution visible only at the subgrain boundaries within certain grains of the Al layer.

The recrystallization distribution map in Figure 5c reveals that both the Cu and Al layers exclusively consist of recrystallized grains or substructures, with deformed grains completely eliminated. A comprehensive analysis of Figure 5a–c elucidate the different origins of substructured grains in the Cu and Al layers. For the Al layer, substructured grains primarily arise from subgrain boundaries containing high-density dislocations within the grains, indicative of incomplete recrystallization. In contrast, for the Cu layer, the low stacking-fault energy of Cu facilitates the formation of a substantial number of twins during annealing [21]. These twins generate large misorientations with adjacent grains, thereby giving rise to substructured grains. The statistical results presented in Figure 5d indicates that in the Al layer, recrystallized grains account for 72%, substructures for 27.49%, and deformed grains account for 0.51%. In the Cu layer, the corresponding proportions are 90.81% for recrystallized grains, 9.1% for substructures, and 0.09% for deformed grains. During high-temperature annealing, merging of grains and growth occur in the Al layer, resulting in the formation of substructured grains and decrease in recrystallized grains. Compared to the 300 °C-annealed samples (Figure 4d), the recrystallization degree of the Cu layer is enhanced due to the more input of thermal energy.

### 3.2. Tensile Properties of Cryorolled and Hot-Rolled Samples

**Figure 6:**
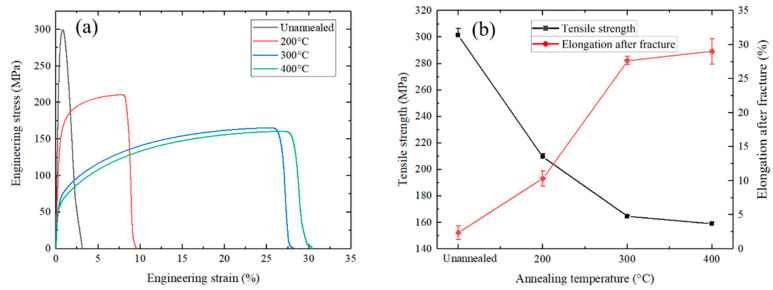
Tensile properties of the Cu/Al clad sheets annealed at different temperatures: (**a**) engineered stress–strain curves, and (**b**) tensile strength and elongation after fracture varies with annealing temperature.

Figure 6 presents the tensile properties of the cryorolled Cu/Al clad sheet with an SUS304 interlayer after annealing. The overall trend is that as the annealing temperature increases, the strength of the clad sheet gradually decreases, while its elongation progressively increases. The tensile strength and elongation of the unannealed specimen are 302 (±3) MPa and 2.4 (±1.5)%, respectively. After annealing at 200 °C, the strength of the specimen decreases to 209 (±1.5) Mpa, while the plasticity increases to 10.3 (±1.7)%. Following annealing at 300 °C, the tensile strength and elongation of the specimen are 165 (±0.8) MPa and 27.6% (±0.5), respectively. For the 400 °C-annealed sample, the tensile strength and elongation are 159 (±0.6) MPa and 29.0 (±2.8)%, respectively.

**Figure 7:**
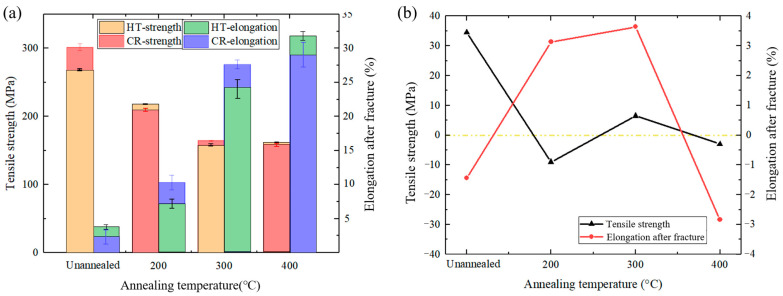
Tensile properties of cryorolled and hot-rolled samples annealed at different temperatures: (**a**) tensile strength and elongation after fracture, and (**b**) difference in tensile properties.

Figure 7 compares the tensile properties of cryorolled and hot-rolled clad sheets. In the unannealed state, the cryorolled specimen exhibits a higher tensile strength compared to the hot-rolled counterpart, albeit with a slightly lower elongation. Specifically, the differences in tensile strength and elongation are 35 MPa and 1.44%, respectively. Upon annealing at 200 °C, the cryorolled specimen undergoes a more pronounced effect from annealing, resulting in a narrower gap with the hot-rolled specimen in terms of tensile strength (9 MPa) and a superior elongation (3.13%). After annealing at 300 °C, the tensile strengths of both cryorolled and hot-rolled specimens become nearly equivalent, while the cryorolled specimen exhibits a marginally higher elongation. When the annealing temperature escalates to 400 °C, the tensile strengths of both specimens remain comparable, but the hot-rolled specimen demonstrates a greater elongation, with disparities of 3 MPa and 2.83% in tensile strength and elongation.

In summary, cryorolling enhances the tensile strength of the clad sheet in the unannealed state. The stress concentration induced by severe plastic deformation leads to the relatively low elongation [19]. As the annealing temperature increases, the distinction in tensile strength between cryorolled and hot-rolled specimens diminishes. At annealing temperatures of 200 °C and 300 °C, the two processes yield comparable results, with cryorolling marginally improving the elongation.

### 3.3. Effect of Cryorolling on Tensile Strength

**Figure 8:**
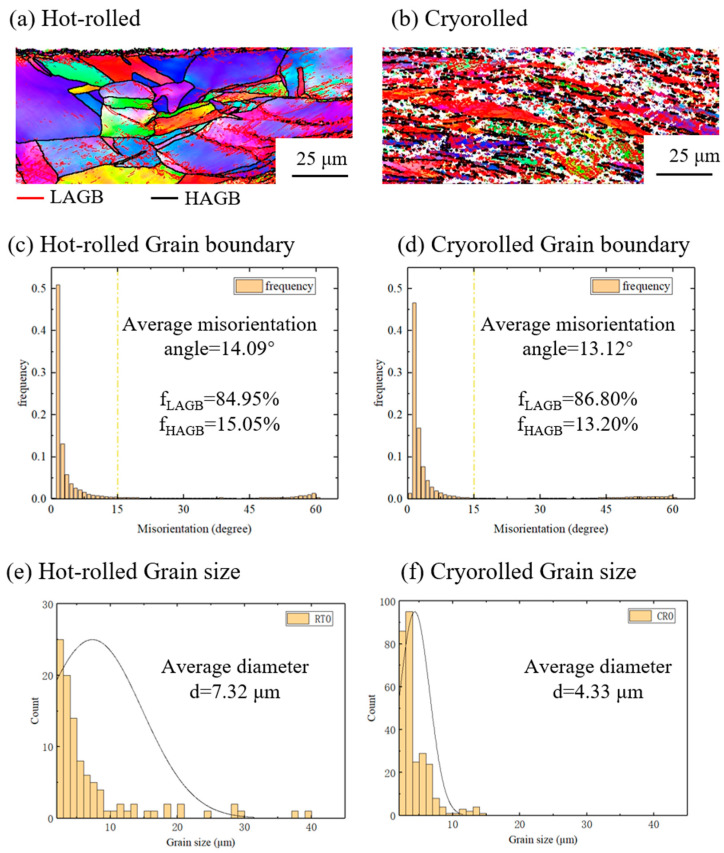
Comparison between cryorolled samples and hot-rolled samples: (**a**,**b**) IPF images, (**c**,**d**) misorientation angle of grain boundaries, and (**e**,**f**) distribution of grain size.

According to the aforementioned results, the cryorolled specimens exhibit a superior tensile strength of 302 MPa, representing a 35 MPa enhancement over hot-rolled samples, which translates to a 13.1% increase in tensile strength within a 70% rolling reduction. Due to the high stack-fault energy [22,23], cryorolling temperature has almost no effect on the microstructure and properties of the Al layer. Our previous research [17] indicated that the SUS304 interlayer can significantly strengthen the bonding strength. The enhanced interfacial bonding strength promoted the coordinated deformation of Cu and Al layers and the improvement of the mechanical properties of Cu/Al clad sheets [24]. Nevertheless, the SUS304 interlayer fractured at the bonding interface during the roll-bonding process, which has little impact on the tensile properties. Therefore, a comparative microstructural analysis was conducted on the Cu layer to further elucidate the mechanism underlying the enhanced tensile strength imparted by cryorolling, as presented in Figure 8.

The analysis of grain boundary misorientations in Figure 8c,d reveal that for hot-rolled specimens, the proportion of low-angle grain boundaries (LAGBs) is 84.95%, while high-angle grain boundaries (HAGBs) account for 15.05%, resulting in an average grain boundary misorientation angle of 14.09°. In contrast, cryorolled specimens exhibit an increased LAGB proportion of 86.80% and a reduced HAGB proportion of 13.20%, with an average grain boundary misorientation angle of 13.12°. Moreover, Figure 8e,f demonstrate that the average grain size of hot-rolled specimens is 7.32 µm, whereas that of cryorolled specimens is refined to 4.33 µm. The ability of cryorolling to promote a higher proportion of LAGBs, coupled with the prevalence of numerous LAGBs within grains as shown in Figure 8b, indicates that cryorolling facilitates microstructural refinement, which underpins the overall strength enhancement of the clad sheet.

Previous study has confirmed that the cryogenic pre-strain could enable a higher density of dislocations within grains, thereby enhancing tensile strength [25]. Similarly, Yu et al. [26] achieved a more refined microstructure in cryorolled Al/Ti/Al clad sheets, attributable to the inhibition function of the cryogenic environment on the dynamic recovery, which resulted in higher tensile strength compared to hot-rolled samples.

### 3.4. Effect of Cryorolling on the Elongation after Fracture

In the comparative analysis of tensile properties between cryorolled and hot-rolled specimens presented in Figure 7, it is observed that cryorolling not only enhances the tensile strength of unannealed samples but also improves the elongation after fracture to a certain extent after annealing at elevated temperatures while maintaining a tensile strength comparable to hot-rolled samples. Similar trends have been identified by Chang et al. [27] in Cu/Al clad sheets processed by asymmetric rolling, where higher deformation levels exhibited relatively higher elongations after fracture post-annealing.

**Figure 9:**
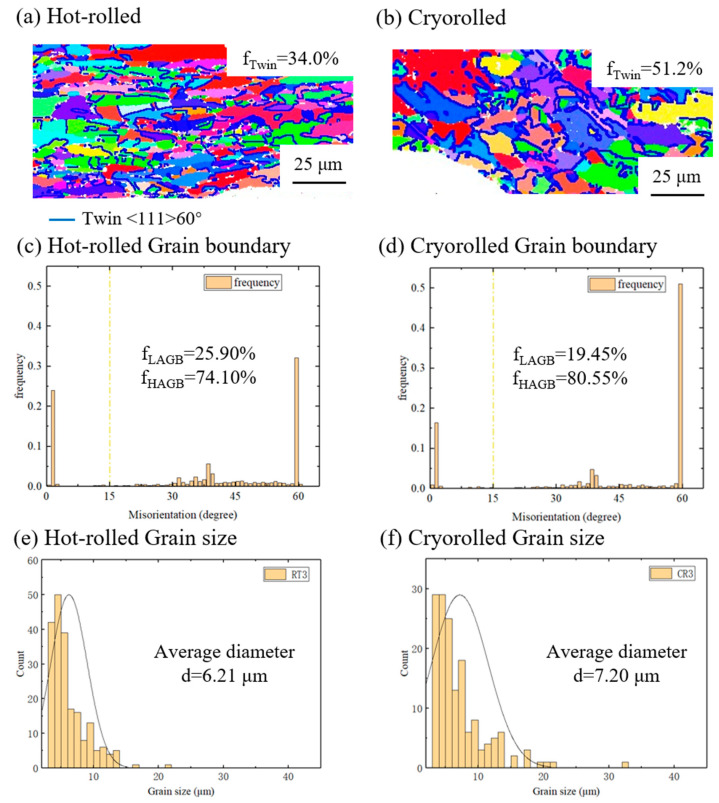
Comparison between cryorolled and hot-rolled samples subjected to 300 °C annealing: (**a**,**b**) IPF images, (**c**,**d**) misorientation angle of grain boundaries, and (**e**,**f**) distribution of grain size.

Figure 9 illustrates the microstructural contrast on the Cu layer of cryorolled and hot-rolled Cu/Al clad sheets after annealing at 300 °C. The Al layer undergoes near-complete recrystallization at 300 °C, with the primary microstructural differences manifesting on the Cu layer. For hot-rolled samples with an average grain size of approximately 6.21 µm, the proportion of LAGBs is 25.90% and that of HAGBs is 74.10%. In contrast, cryorolled samples exhibit an average grain size of about 7.20 µm, accompanied by a LAGB proportion of 19.45% and an HAGB proportion of 80.55%. Notably, cryorolled samples demonstrate higher HAGB proportions and grain sizes than hot-rolled ones, indicative of a higher degree of recovery post-annealing at 300 °C, which contributes to their superior elongation after fracture. The comparable tensile strength between cryorolled and hot-rolled samples is primarily attributed to the formation of annealing twins, with a proportion of 51.2% in cryorolled samples, exceeding the 34.0% observed in hot-rolled samples. Li et al. [28] also reported similar findings in the microstructural study of asynchronously rolled Cu/Al clad sheets after annealing, where numerous annealing twins were observed within the recovered Cu layer, reflecting the accumulation of significant strain energy.

## 4. Conclusions

In this work, the microstructure evolution of cryorolled Cu/Al clad sheet with an SUS304 interlayer after annealing at various temperatures are investigated. Furthermore, the tensile properties of cryorolled and hot-rolled samples are compared. The main conclusions are as follows:

(1) Benefiting from the significant grain refinement effect of cryorolling, the tensile strength of the cryorolled samples reached as high as 302 MPa with the elongation after a fracture of 2.4%. After annealing, the proportion of recrystallized grains and substructured grains gradually increased. Upon annealing at 400 °C, the tensile strength decreased to 159 MPa, while the elongation recovered to 29.01%.
(2) The ability of cryorolling to promote a higher proportion of LAGBs, coupled with the prevalence of numerous LAGBs within grains enhances the microstructural refinement, which is 35 MPa higher in tensile strength than that of the hot-rolled samples. A 13.1% increase is achieved within a total of 70% rolling reduction.
(3) For samples annealed at 300 °C, the cryorolled samples exhibit a higher degree of recrystallization than the hot-rolled samples, resulting in an improved elongation after fracture. The strength comparable to the hot-rolled clad sheet is primarily attributed to the higher proportion of annealing twins.

## Figures and Tables

**Table 1:** Mechanical properties of Cu, Al, and SUS304 foil.

Materials	Tensile Strength/MPa	Elongation after Fracture/%
T2 Cu	205	46
1060 Al	70	56
SUS304	1318	10.2

## Data Availability

The original contributions presented in the study are included in the article, further inquiries can be directed to the corresponding author.

## References

[1] Han J.C., Li S., Gao X.Y., Huang Z.Q., Wang T., Huang Q.X. (2023). Effect of annealing process on interface microstructure and mechanical property of the Cu/Al corrugated clad sheet. J. Mater. Res. Technol..

[2] Xu R.Z., Li F.S., Yuan C.C., Zhang Y., Yan W.D., Zhao X. (2021). Microstructure and phase constitution at the interface of double-sided electron beam welded Cu/Al clad metal sheet. Mater. Charact..

[3] Wang Y.J., Song R.B., Yanagimoto J., Li H. (2019). Effect of heat treatment on bonding mechanism and mechanical properties of high strength Cu/Al/Cu clad composite. J. Alloys Compd..

[4] Gholami M.D., Hashemi R., Davoodi B. (2023). Investigation of microstructure evolution on the fracture toughness behaviour of brass/low carbon steel/brass clad sheets fabricated by cold roll bonding process. J. Mater. Res. Technol..

[5] Mao Z.P., Xie J.P., Wang A.Q., Wang W.Y., Ma D.Q., Liu P. (2020). Effects of annealing temperature on the interfacial microstructure and bonding strength of Cu/Al clad sheets produced by twin-roll casting and rolling. J. Mater. Process. Technol..

[6] Li Z.H., Yang Y., Liang X.P., Zhang W.D., Cao L.M., Wu C.P., Zeng Z.H., Wang L. (2020). Effect of annealing temperature and time on the microstructure, mechanical properties and conductivity of cold-rolled explosive Cu/Al composite sheets. Mater. Res. Express.

[7] Xu J.M., Gao X., Dou H., Zhang C.P., Wang W., Liu K. (2023). Improved cryogenic frictional properties of thrust ball bearings in liquid nitrogen through PTFE cages and dimple-type textures. Cryogenics.

[8] Wang Z.J., Ma M., Qiu Z.X., Zhang J.X., Liu W.C. (2018). Microstructure, texture and mechanical properties of AA1060 aluminum alloy processed by cryogenic accumulative roll bonding. Mater. Charact..

[9] Takagawa Y., Tsujiuchi Y., Watanabe C., Monzen R., Tsuji N. (2013). Improvement in mechanical properties of a Cu-2.0 mass% Ni-0.5 mass% Si-0.1 mass% Zr alloy by combining both accumulative roll-bonding and cryo-rolling with aging. Mater. Trans..

[10] Liu J., Wu Y.Z., Wang L., Kong C., Pesin A., Zhilyaev A.P., Yu H.L. (2020). Fabrication and characterization of high bonding strength Al/Ti/Al-laminated composites via cryorolling. Acta Metall. Sin..

[11] Macwan A., Jiang X.Q., Li C., Chen D.L. (2013). Effect of annealing on interface microstructures and tensile properties of rolled Al/Mg/Al tri-layer clad sheets. Mater. Sci. Eng. A.

[12] Kim J.S., Lee K.S., Kwon Y.N., Lee B.J., Chang Y.W., Lee S. (2015). Improvement of interfacial bonding strength in roll-bonded Mg/Al clad sheets through annealing and secondary rolling process. Mater. Sci. Eng. A.

[13] Sung H.M., Lee S., Lee D., Kim H., Kang S.G., Lee G.D., Jeong K., Han N.H. (2024). Effect of the Ni plating on Al-Cu dissimilar metal laser welded joint. J. Mater. Res. Technol..

[14] Tayyebi M., Alizadeh M., Lech S. (2024). Characterizing of a unique Al/Cu FGMMC fabricated via the ARB-CRB process followed by annealing. J. Alloys Compd..

[15] Pelzer R., Nelhiebel M., Zink R., Woehlert S., Lassnig A., Khatibi G. (2012). High temperature storage reliability investigation of the Al-Cu wire bond interface. Microelectron. Reliab..

[16] Lee W.B., Bang K.S., Jung S.B. (2005). Effects of intermetallic compound on the electrical and mechanical properties of friction welded Cu/Al bimetallic joints during annealing. J. Alloys Compd..

[17] Gao H.T., Wang L., Liu S.L., Li J., Kong C., Yu H.L. (2021). Effects of a stainless steel interlayer on the interfacial microstructure and bonding strength of Cu/Al clad sheets prepared via the powder-in-tube method. J. Mater. Res. Technol..

[18] (2016). Metallic materials—Tensile testing.

[19] Liu Y., Xu J., Ning T., Yang H.N., Tan Z.G., Guo S.B., Wang M.B. (2024). Process optimization and microstructure evolution of Inconel718 alloy by laser power bed fusion. China Powder Sci. Technol..

[20] Sheng L.Y., Yang F., Xi T.F., Lai C., Ye H.Q. (2011). Influence of heat treatment on interface of Cu/Al bimetal composite fabricated by cold rolling. Compos. Part B.

[21] San X.Y., Liang X.G., Cheng L.P., Li S., Zhu X.K. (2012). Effect of stacking fault energy on mechanical properties of ultrafine-grain Cu and Cu-Al alloy processed by cold-rolling. Trans. Nonferr. Met. Soc. China.

[22] Xue F., Fan Z., Chen Y., Li J., Wang H., Zhang X. (2015). The formation mechanisms of growth twins in polycrystalline Al with high stacking fault energy. Acta Mater..

[23] Xue S., Kuo W., Li Q., Fan Z., Ding J., Su R., Wang H., Zhang X. (2018). Texture-directed twin formation propensity in Al with high stacking fault energy. Acta Mater..

[24] Tang B.B., Feng S.Y., Duan J.Y., Jin P.P. (2024). Preparation and interfacial behavior of particle-reinforced aluminum matrix composites. China Powder Sci. Technol..

[25] Wu S.S., Xin J.J., Xie W., Zhang H.C., Huang C.J., Wang W., Zhou Z.R., Zhou Y., Li L.F. (2022). Mechanical properties and microstructure evolution of cryogenic pre-strained 316LN stainless steel. Cryogenics.

[26] Yu H.L., Lu C., Tieu K., Li H.J., Godbole A., Liu X., Kong C. (2017). Enhanced materials performance of Al/Ti/Al laminate sheets subjected to cryogenic roll bonding. J. Mater. Res..

[27] Chang D.X., Wang P., Zhao Y.Y. (2020). Effects of asymmetry and annealing on interfacial microstructure and mechanical properties of Cu/Al laminated composite fabricated by asymmetrical roll bonding. J. Alloys Compd..

[28] Li X.B., Zu G.Y., Wang P. (2015). Microstructural development and its effects on mechanical properties of Al/Cu laminated composite. Trans. Nonferr. Met. Soc. China.

